# Epidemiological and Molecular Characteristics of *Piroplasmids* and *Anaplasma* spp. in Tan Sheep, Ningxia, Northwest China

**DOI:** 10.1155/2024/2529855

**Published:** 2024-05-29

**Authors:** Jiali Zhou, Zhixin Li, Zicheng Zhou, Yue Ma, Junhao Hu, Xingang Dan, Hongxi Zhao

**Affiliations:** ^1^ College of Animal Science and Technology Ningxia University Yinchuan 750000China; ^2^ Ningxia Animal Disease Prevention and Control Center Yinchuan Ningxia 750011China

## Abstract

Piroplasmosis and anaplasmosis are important zoonotic diseases of animal origin, which can be transmitted by ticks to infect animals. However, there is limited information on the infection of piroplasmosis and anaplasmosis in Tan sheep in Ningxia, China. In order to understand the prevalence of piroplasmosis and anaplasmosis in Tan sheep in Ningxia, 150 blood samples of Tan sheep from farms in five urban areas of Ningxia were detected by PCR, and some positive samples were sequenced to establish a phylogenetic tree. PCR revealed that the prevalence of *Anaplasma* spp. in Tan sheep in Ningxia was 28.0%. The overall prevalence of *Piroplasmids* was 33.3%, of which *Theileria* spp. and *Babesia* spp. were 20.7% and 12.7%, respectively. Among the samples of different ages, the highest detection rates of *Piroplasmids* and *Anaplasma* spp. were found in Tan sheep aged 20–30 months, and the detection rate of *Theileria* spp., *Babesia* spp., and *Anaplasma* spp. were 25.4%, 23.6%, and 36.3%, respectively. In this study, one *Theileria* species was identified as *Theileria uilenbergi*, two *Babesia* species were identified as *Babesia molasi* and *Babesia ovis*, and two *Anaplasma* species were identified as *Anaplasma ovis* and *Anaplasma phagocytophilum*, and the dominant species were *A. ovis* and *T. uilenbergi*. To the best of our knowledge, this is the first report detailing the infection rate and genotype of *Piroplasmids* and *Anaplasma* spp. in Tan sheep in Ningxia, China. The results of this study provide valuable data for understanding the epidemiology of tick-borne disease in Tan sheep in Ningxia, China, and lay a theoretical foundation for the prevention and control of piroplasmosis and anaplasmosis in Tan sheep in Ningxia, northwest China.

## 1. Introduction

As an obligate blood-sucking ectoparasite, ticks are the second largest insect vector after mosquitoes [1], which can act as a vector for the transmission of variety of pathogens including bacteria, fungi, viruses, and protozoa [2]. These organisms can cause many important emerging or re-emerging infectious diseases, such as severe fever with thrombocytopenia syndrome [3], human granulocytic anaplasmosis [4], and Crimean–Congo hemorrhagic fever [5], causing great harm to human and animal health worldwide [6, 7, 8]. However, piroplasmosis and anaplasmosis are common tick-borne diseases in cattle and sheep. Piroplasmosis is a tick-borne protozoan disease caused by *Babesia* and *Theileria* in ruminants [9]. Among them, babesiosis of sheep is a blood parasitic disease characterized by anemia, emaciation, and jaundice caused by different species of *Babesia* species parasitic in mammalian erythrocytes [10]. It is reported that eight species of *Babesia* spp. have been found in horses, cattle, sheep, and dogs in China. Five species of *Babesia* spp. have been reported to infect sheep, *B. motasi*, *B. ovis*, *Babesia crassa*, *Babesia taylori*, and *Babesia foliata* [11]. Among them, *B. ovis* infection is the most serious blood parasitic disease, with clinical manifestations including fever, hemoglobinuria, hemolytic anemia, and jaundice, resulting in the death of 30%–50% of infected sheep worldwide [12, 13]. Theileriosis is a blood protozoal disease that is transmitted to sheep by the protozoa of the genus *Theileria* through tick-infected red blood cells, macrophages and lymphocytes. The disease is characterized by high fever, superficial lymphadenopathy, anemia, anorexia, lethargy, respiratory distress, and death, and Giemsa-stained blood smears showing polymorphic erythrocytes parasitized by *Theileria* [14, 15]. In recent years, *Theileria luwenshuni*, *Theileria uilenbergi*, and *Theileria ovis* have been reported to infect sheep in China [16, 17, 18]. *Anaplasma* belongs to Rickettsiales Anaplasmataceae, which is an obligate and intracellular parasitic Gram-negative bacteria. It is mainly transmitted by ticks and can infect a variety of intracellular parasitic pathogens in animals and humans [19]. At present, the identified species include *Anaplasma bovis*, *A. phagocytophilum*, and *A. ovis* [20]. The clinical symptoms of sheep infected with the above pathogens are similar, and all of them can be transmitted by ticks. With the rapid development of the sheep industry in China, exchanges of sheep and lamb products in different regions have become more frequent, and the epidemic areas of sheep piroplasmosis and anaplasmosis have gradually increased in China [21, 22].

Theileriosis, babesiosis, and anaplasmosis are all transmitted by ticks in sheep, and mixed infection is easy to occur. The morphology of the three pathogens is similar in red blood cells, and it is not easy to distinguish them by blood smear, but PCR can be used for differential diagnosis. Tan sheep, one of the sheep breeds in Ningxia, mainly distributed in the central region of Ningxia, is the main pillar industry of animal husbandry in some places. Because of its tender meat and delicious taste, it has become an important means for local farmers to increase their income. Piroplasmosis and anaplasmosis have seriously affected the healthy development of the Tan sheep industry, but there are no related data on *Piroplasmids* and *Anaplasma* spp. in Ningxia Tan sheep. In this study, 150 blood samples of Tan sheep from farmers in five regions of Ningxia Hui Autonomous Region were collected, and *Theileria*, *Babesia*, and *Anaplasma* were detected by PCR. This study aimed to investigate the prevalence and genetic diversity of *Piroplasmids* and *Anaplasma* spp. infections in Tan sheep in Ningxia Hui Autonomous Region and to lay a theoretical foundation for the prevention and control of *Piroplasmids* and *Anaplasma* spp. infections in Tan sheep in Ningxia.

## 2. Materials and Methods

### 2.1. Sample Collection

**Figure 1:**
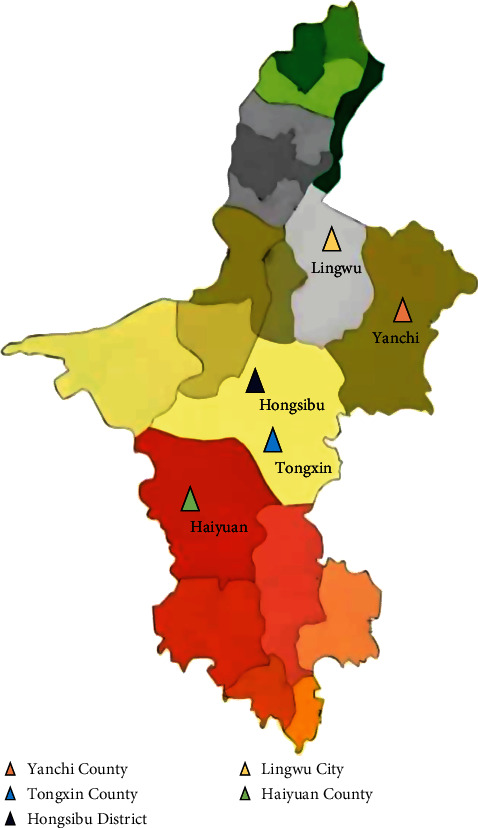
Map of the sampling locations in Ningxia, China.

From June 3 to 9, 2023, blood samples were randomly collected from 150 Tan sheep with similar body weight and clinical manifestations of fever (the body temperature exceeds 40°C), emaciation, and jaundice in Yanchi County, Haiyuan County, Tongxin County, Lingwu City, and Hongsibu District of Ningxia Hui Autonomous Region. 30 samples from each region (Figure 1). Blood samples were collected aseptically from the jugular vein of Tan sheep. These samples were immediately placed into EDTA anticoagulated tubes. Concurrently, detailed information such as the time and location of collection, as well as the age of the sheep, was recorded. Samples were stored in ice boxes after collection and stored at 4°C in the laboratory until screening.

### 2.2. Extraction of Genomic DNA

Following the instructions form the whole blood genomic DNA extraction kit by Tiagen Biochemical Technology (Beijing) Co.Ltd., sheep blood samples were extracted, and then, the DNA was stored at −20°C. It was used for PCR amplification of target genes.

### 2.3. PCR Detection of *Piroplasmids* and *Anaplasma* spp.

#### 2.3.1. PCR detection of *Theileria* and *Babesia* in Tan Sheep

According to the 18S rRNA gene sequence of *Theileria* spp., specific primers for *T. luwenshuni*, *T. uilenbergi*, and *T. ovis* were synthesized, respectively. According to the 18S rRNA gene sequence of *Babesia* spp., specific primers for *B. ovis* and *B. motasi* were designed, respectively. All primers were synthesized by BioEngineering (Shanghai) Co.Ltd. *Theileria* and *Babesia* were amplified from the blood samples of Tan sheep by PCR, and the PCR products were electrophoresed in 1% (w/v) agarose gel, and the results were observed under a gel imager.

#### 2.3.2. PCR Detection of *Anaplasma* spp.

According to the 16S rRNA gene sequence of *A. phagocytophilum* and *A. bovis*, specific primers for *A. phagocytophilum* and *A. bovis* were synthesized, respectively, and specific primers for *A. ovis* msp4 gene were used for PCR amplification. The primers were synthesized by BioEngineering (Shanghai) Co.Ltd. PCR products were electrophoresed in a 1% (w/v) agarose gel, and the results were visualized under a gel imager. The sequences of each primer are shown in Table 1.

### 2.4. Phylogenetic Analysis

Positive PCR products were sent to BioEngineering (Shanghai) Co.Ltd. for sequencing. After shearing the sequencing results, blast alignment in NCBI revealed sequences with high similarity to the target fragment. Phylogenetic tree analysis was performed using MEGA 7.0 software.

## 3. Results

### 3.1. Detection Rates of *Piroplasmids* and *Anaplasma* spp. in Tan Sheep in Different Regions

A total of 150 blood samples were detected by PCR from five regions in Ningxia (Table 2). The highest infection rate of *Theileria* spp. was found in Yanchi County at 30.0% (10/30), followed by Hongsibu County with 26.7% (8/30), Lingwu City with 23.3% (7/30), Haiyuan County with 13.3% (4/30), and Tongxin County with the lowest rate at 6.0% (2/30). The infection rates of *Babesia* spp. were 16.7% (5/30), 16.7% (5/30), and 30% (9/30) in Yanchi County, Lingwu City, and Hongsibu County, respectively, and none was detected in Haiyuan County and Tongxin County, and Hongsibu County had the highest infection rate of *Babesia*. The infection rate of *Anaplasma* spp. was 46.7% (14/30) in Yanchi County, 26.7% (8/30) in Haiyuan County, 20% (6/30) in Tongxin County, 3.3% (1/30) in Lingwu City, and 43.3% (13/30) in Hongsibu County, respectively. The highest infection rate of *Anaplasma* spp. was in Yanchi County, followed by Hongsibao County.

The detection of different species of *Piroplasmids* and *Anaplasma* spp. in five areas of Ningxia showed that among the infected *Theileria* spp., the dominant species of infection was *T. uilenbergi*, but *T. luwenshuni* and *T. ovis* were not detected (Table 3). Among infected *Babesia* spp., the detection rates of *B. ovis and B. molasi* were similar. Among the infected *Anaplasma* spp., the highest detection rate was *A. ovis*, while the detection rate of *A. phagocytophilum* was relatively low. Totally, 150 blood samples were collected from Tan sheep, and *Theileria* spp. were detected from 31 blood samples (20.7%); *Babesia* spp. were detected from 19 blood samples (12.7%); *Anaplasma* spp. were detected from 42 blood samples (28%); and 7 blood samples (4.7%) were mixed infection.

### 3.2. Age Distribution of *Piroplasmids* and *Anaplasma* spp. in Tan Sheep

According to Table 4, statistics on the detection of *Theileria* spp.in Tan sheep of different ages showed that the highest detection rate was 25.4% in sheep aged 20–30 months, while the lowest detection rate was 12.2% in sheep aged 30–40 months. A significant difference in the infection rate of *Theileria* spp. was among sheep of different age groups (*P* < 0.05). The highest detection rate of *Babesia* spp. was 23.6% in sheep aged 20–30 months, which was significantly different from the rates in those aged 30–40 months and 40–50 months (*P*  < 0.05). The highest detection rate of *Anaplasma* spp. was 36.3% in the 20–30 months old group, while the lowest was 18.3% in the 30–40 months old group, indicating a significant difference in detection rates across age groups (*P*  < 0.05).

### 3.3. Sequence Analysis and Establishment of Phylogenetic Trees

**Figure 2:**
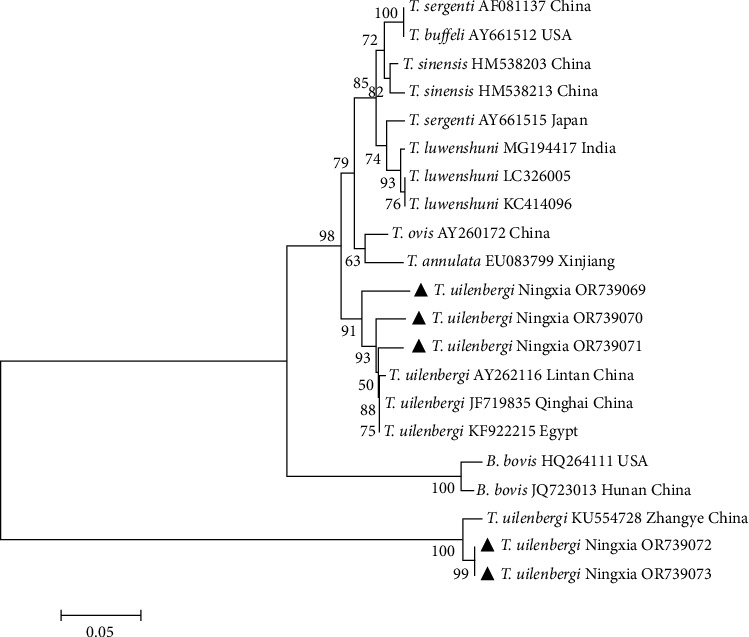
Phylogenetic tree of 18S rRNA nucleotide sequences of *Theileria uilenbergi*. *Note*. The sequences obtained in this study are marked with black triangle symbols. Phylogenetic analyses were performed using the maximum-likelihood method in MEGA7.0, the Kimura 2-parameter model, and 1,000 bootstrapping.

The 18S rRNA gene sequences of five *T. uilenbergi* isolates identified by sequencing were submitted to GenBank, and the sequence accession numbers were OR739069–OR739073. From the constructed phylogenetic tree (Figure 2), the five isolates of *T. uilenbergi* from Ningxia, China, were divided into two groups. *T. uilenbergi* Ningxia (OR739069–OR739071) belonged to the same clade with *T. uilenbergi* isolates from Lintan (AY262116), Qinghai (JF719835), and Egypt (KF922215). *T. uilenbergi* Ningxia (OR739072–OR739073) and *T. uilenbergi* (KU554728) isolated from Zhangye, China, were in the same branch, and the similarity reached 99%. In addition, the five *T. uilenbergi* isolates were not closely related to *T. luwenshuni* and *T. ovis* prevalent in China, and the dominant *T. uilenbergi* strain was found in Tan sheep in some areas of Ningxia.

**Figure 3:**
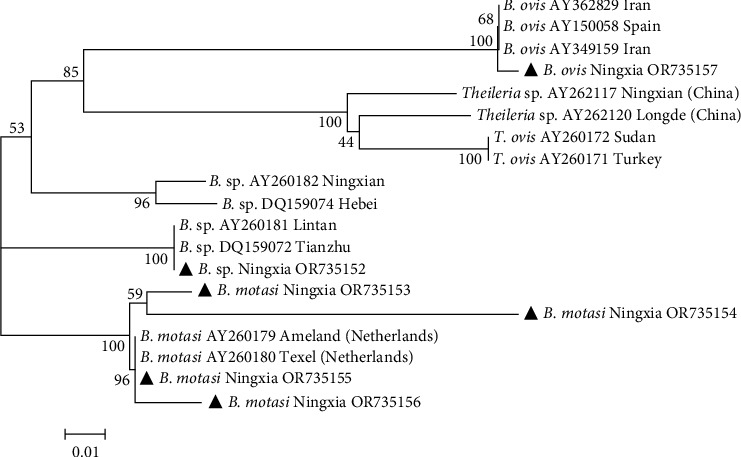
Phylogenetic tree based on *Babesia* spp.18S rRNA nucleotide sequences. *Note*. The sequences obtained in this study are marked with black triangle symbols. Phylogenetic analyses were performed using the maximum-likelihood method in MEGA7.0, the Kimura 2-parameter model, and 1,000 bootstrapping.

The 18S rRNA gene sequences of six *Babesia* spp. isolates identified by sequencing were submitted to GenBank, and the sequence accession numbers were OR735152–OR735157. From the constructed phylogenetic tree (Figure 3), the six Chinese isolates of *Babesia*. spp. Ningxia were divided into three groups. The *B. ovis* isolates from Iran (AY362829, AY349159) and Spain (AY150058) shared 100% similarity with *B. ovis* Ningxia (OR735157). The *Babesia* sp. isolates from Lintan (AY260181) and Tianzhu (DQ159072) shared 100% similarity with *B*. sp. Ningxia (OR735152). And *B. motasi* Ningxia (OR735153-OR735156) shared 99% similarity with *B. motasi* isolates from the Netherlands (AY260179, AY260180).

**Figure 4:**
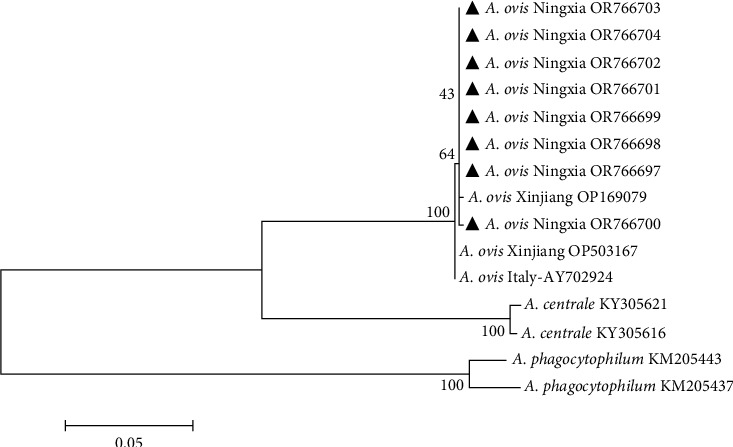
Phylogenetic tree of *Anaplasma ovis* msp4 gene. *Note*. The sequences obtained in this study are marked with black triangle symbols. Phylogenetic analyses were performed using the maximum-likelihood method in MEGA7.0, the Kimura 2-parameter model, and 1,000 bootstrapping.

The msp4 gene sequences of eight *A. ovis* strains identified by sequencing were submitted to GenBank, and the sequence accession numbers were OR766697–OR766704. From the constructed phylogenetic tree (Figure 4), the *A. ovis* isolates from Italy (AY702924) and Xinjiang (OP503167, OP169079) had 100% similarity with *A. ovis* Ningxia (OR766697–OR766704). The other two groups were *A. centrale* and *A. phagocytophilum*, which were not in the same branch as *A. ovis* isolated from Tan sheep in Ningxia and other *A. ovis* isolated from domestic and foreign areas, which also indicated that all *A. ovis* strains isolated in this study were *A. ovis*.

**Figure 5:**
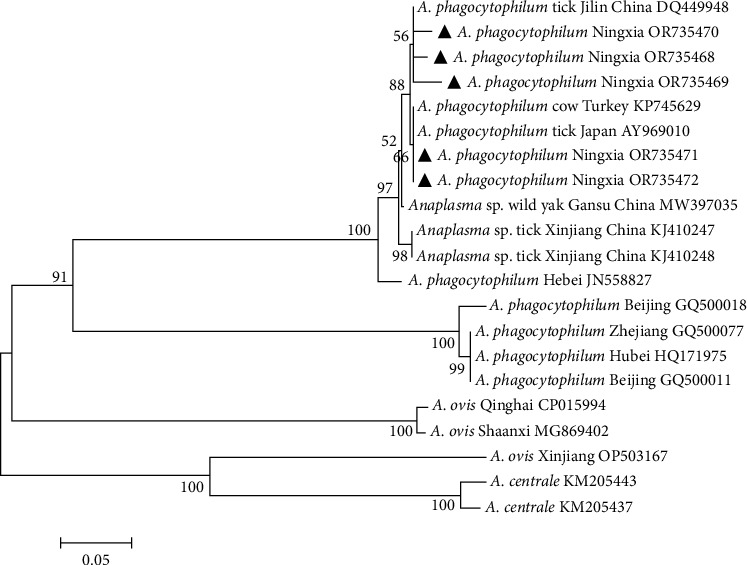
Phylogenetic tree based on 16S rRNA nucleotide sequences of *Anaplasma phagocytophilum*. *Note*. The sequences obtained in this study are marked with black triangle symbols. Phylogenetic analyses were performed using the maximum-likelihood method in MEGA7.0, the Kimura 2-parameter model, and 1,000 bootstrapping.

The 16S rRNA gene sequences of five *A. phagocytophilum* strains identified by sequencing were submitted to GenBank, and the sequence accession numbers were OR735468–OR735472. According to the phylogenetic tree constructed (Figure 5), the isolated *A. phagocytophilum* pathogens had complex and variable genotypes, including unique local strains in Ningxia and strains with similar genotypes with domestic and foreign strains. Five isolates were closely related to Jilin (DQ449948) and Gansu (MW397035) in China. The isolates from Turkey (KP745629) and Japan (AY969010) shared high similarity. The other two groups were *A. centrale* and *A. ovis*, which were not in the same branch as *A. phagocytophilum* isolated from Tan sheep in Ningxia and *A. phagocytophilum* isolated from other countries at home and abroad, indicating that all *A. phagocytophilum* strains isolated in this study were *A. phagocytophilum*.

## 4. Discussion

In recent years, many articles have reported the prevalence of sheep infected with *Piroplasmids* and *Anaplasma* spp. in China, ranging from 15% to 50% [2, 22, 26, 27, 28]. Li et al. [29] nested polymerase chain reaction (nPCR) assays and gene sequencing were used to identify tick-borne *Babesia* spp., *Theileria* spp., and *Anaplasma* spp. infections in border regions, northwestern China. Out of 323 samples tested in this study, 225 (69.7%) sheep were infected with *Babesia* spp., *Theileria* spp., and *Anaplasma* spp. Two hundred six (63.8%), 60 (18.6%), 54 (16.7%), 51 (15.8%), 32 (9.9%), 19 (5.9%), and 16 (5.0%) were positive for *A. ovis*, *B. motasi-like*, *A. bovis*, *T. uilenbergi*, *A. phagocytophilum*, *T. luwenshuni*, and *B. motasi*-like Xinjiang, respectively. Molecular detection of tick-borne pathogens harbored by ticks collected from livestock in the Xinjiang Uygur Autonomous Region, China, founding the most common pathogen was *Rickettsia raoultii* (36.80%), followed by *Brucella* sp. (26.2%), *A. ovis* (22.4%), *Babesia caballi* (14.8%), *Theileria equi* (8.7%), and *T. ovis* (8.5%) [30]. In addition, El-Alfy et al. [31] conducted a systematic review and meta-analysis of the molecular epidemiology and species diversity of tick-borne pathogens in animals in Egypt and found that the infection rates of tick-borne pathogens (TBPs), *Babesia* spp., and *Theileria* spp. in sheep and goats were 3.8% and 11.0%, respectively. Meanwhile, diverse faunas of TBPs were identified in sheep, including various species of the genus *Babesia* (*B. bovis*, *B. bigemina*, *and B. ovis*), the genus *Theileria* (*T. annulata*, *T. ovis*, *and Theileria lestoquardi*), and the genus *Anaplasma* (*A. marginale*, *A. ovis*, *A. phagocytophilum*, *A. platys*, and *A. platys-like*). In this study, the detection rate of *Theileria* spp. was 20.7%, which was lower than the detection rate reported in most previous studies in China, such as 60.8% in Gansu [32], 50.0% in Hubei [33] and 31.3% in Qinghai [34]. These differences may be related to regions, feeding patterns, and breeds of sheep. The detection rate of *Babesia* spp. was 12.7%, which was slightly higher than that reported in previous major studies in China, such as 3.9% in Xinjiang, 2.0% in Shaanxi, and 1.5% in Gansu [26]. The detection rate of *Anaplasma* spp. was 28%, which is higher than that in some areas of China, such as Henan (4.4%) and Guizhou (2.6%) [22]). This may be related to the increased awareness of disease prevention among local farmers in recent years, and the reduction of grazing times and vector tick distribution during the grazing season. Previous studies have shown that the impact of parasite on young sheep is greater than that on adult sheep and the detection rate is higher. In this study, because the Tan sheep whose blood was collected were all adult sheep, the age difference is small, but the detection rate of *Babesia* in 20–30 months old sheep is higher than older sheep in other months, which may be due to the difference in feeding management and tick control in the area where the samples were collected at this age.

Six species of *Theileria* spp. infesting sheep have been reported and named, including *T. luwenshuni*, *T. uilenbergi*, *T. ovis*, *T. lestoquardi*, *Theileria recondita*, and *Theileria separata*, among which *T. lestoquardi*, *T. luwenshuni*, and *T. uilenbergi* are highly pathogenic and are known as malignant *Theileria* spp., and *T. ovis*, *T. recondita*, and *T. separata* are known as mild-type *Theileria* spp. because of their weak or no obvious pathogenicity [35]. The molecular evidence, genetic characteristics, and phylogenetic analysis of *Theileria* spp. in sheep of Egypt have been reported abroad. The results showed that the detection rate of *T. ovis* in sheep was 4.37%, and the detection rate of *T. lestoquardi* was 0.87% [36]. In addition, Al-Fahdi et al. [37] conducted a molecular survey of livestock *Theileria* spp. genus in four provinces of Oman, and the results showed that the prevalence of *Theileria* spp. was 72.3%, 36.7%, and 2.7% among cattle, sheep, and goats, respectively. Among the reported sheep *Theileria* spp. in China, *T. luwenshuni* is the dominant one. [38]. However, in this study, the 18S rRNA gene sequences of *Theileria* spp. isolated from sheep were examined in Ningxia, China. The results revealed that only *T. uilenbergi* was detected; *T. ovis* and *T. luwenshuni* were not detected. Some studies have shown that most of *T. luwenshuni* and *T. uilenbergi* were mixed infection in northwest China [39]. However, only *T. luwenshuni* exists in central, southeast, and southwest China [33, 40], and some scholars have reported that *T. luwenshuni* can infect goats, sheep, yellow sheep, cattle, yak, roe deer, sika deer, red deer, Mongolian gazelle, and hedgehog [41], which is different from our results. There are many reasons for this, which may be related to the species of ticks in Ningxia. Different *Piroplasmids* are transmitted by different vectors of ticks. In China, the transmission vectors of *T. luwenshuni* and *T. uilenbergi* are *Haemaphysalis qinghaiensis* and *Haemaphysalis longicornis* [39]. It may also be due to the rapid development of sheep breeding and frequent cross-border transport in China and transmission in other regions or abroad. In addition, Ningxia is located in the northwest of China, which has a typical continental climate, long sunshine time, large temperature difference between day and night, and high temperature in summer, which is suitable for the growth and reproduction of ticks. After the bites of ticks on sheep, animals are in a state of insect-carrying immunity for a long time, and it is difficult to be eradicated. The cured animals with *Theileria* spp. disease show growth retardation and decreased milk and meat production, which has a very negative impact on the development of the breeding industry. The high detection rate of *Theileria* spp. infection in this study may be due to the significant seasonal and regional transmission and prevalence of *Theileria* spp. infection in sheep, which is closely related to the distribution of ticks in the local vector, and the time when we collected samples is a high incidence period of *Theileria* spp. infection. Meanwhile, the reason for the high detection rate of *Theileria* spp. is not only due to the wide variety and seasonal prevalence of *Theileria* spp., which can be infected by the bite of a variety of ticks, but also due to the fact that among the blood samples collected in this study, Tan sheep in Tongxin County were vaccinated 2 months ago, while the Tan sheep in other sample collection areas were not vaccinated. This may also be one of the reasons for the high local detection rate of *Theileria* spp. In conclusion, the incidence of *Theileria* spp. in sheep is gradually increasing, and mild or inapparent infection is common in natural cases, which increases the difficulty of prevention and control of this disease. In Ningxia, especially in the areas with a high detection rate of *Theileria* spp., it is necessary to do a good job of prevention and early diagnosis. Tick control should be carried out on the sheep's surface and pens in spring to autumn, and blood samples should be collected to screen positive cases through early diagnosis and treat them timely, so as to minimize the economic loss of herdsmen.

Babesiosis is an emerging parasitic disease, which is endemic in the world, including Europe, Asia, Africa, North and South America, and Australia. The United States has the largest number of babesiosis cases. Babesiosis is mainly distributed in northeast China, followed by southwest China [42, 43]. According to the current study, of the five *Babesia* spp. known to infect sheep and goats, *B. motasi* and *B. crassa* can infect large animals, and *B. ovis*, *B. taylori*, and *B. folie* can infect small animals [44, 45]. In this study, the 18S rRNA gene sequence of a *B. ovis* isolate from the northern part of sheep was detected in Ningxia, China. Meanwhile, *B. ovis* and *B. motasi* were detected with the infection rates of 6.7% and 6.0%, respectively. Relevant studies have shown that *B. ovis* is mainly distributed in Europe, Africa, Asia, and the Far East. In recent years, studies have shown the prevalence of *Babesia* spp. infection among sheep in different countries and regions, with rates ranging from 5.85% to 86.4% [46, 47]. *B. motasi* originated from southern Europe, and the infection rate of *B. motasi* is 16.7% in the northwest border area of China [29]. Compared with previous studies, the infection rate of *Babesia* spp. was lower in Ningxia, which might be related to the effective prevention and control of *Babesia* spp. in region in recent years. The genotype of *Babesia* spp. isolated in Ningxia had 100% similarity with the isolates of *Babesia* Lintan and *Babesia* Tianzhu in China. The genotypes of *B. ovis* and *B. motasi* shared 100% similarity with other countries. There are many reasons for this situation, and we need to further explore. The research of babesiosis in sheep is still a long way to go. China is a big country of sheep raising, and we must pay attention to the prevention and control of babesiosis in sheep in the process of sheep breeding, so as to provide a guarantee for the healthy development of the sheep industry and human health.

*Anaplasma* is obligate intracellular bacteria of cells of hematopoietic origin and is etiological agents of tick-borne diseases of both veterinary and medical interest common in both tropical and temperate regions. The pathogen has a wide range of hosts and can infect humans, domestic animals, and a variety of wild animals, among which infection of various wild animals is an important reservoir host to maintain its long-term existence in nature [48]. There are six kinds of pathogens *A. phagocytophilum*, *A. Marginale*, *A. bovis*, *A. centrale*, *A. ovis*, *and A. capra* [49]. At present, the disease of *Anaplasma* spp. has been found all over the world. For instance, a study by Wei et al. [50] investigated the prevalence of *Anaplasma* spp. infection in goats in Beijing, China. They found that 44.6% (41/92) of goats were infected with *A. capra*, while the 22.8% (21/92) were infected with *Anaplasma*. In addition, in China, the prevalence rates were 19.4%, 19.3%, 10%, 8.8%, 6.8%, 1.8%, and 0% in goats from Guizhou Province, Henan Province, Inner Mongolia Autonomous Region, Shanxi Province, Xinjiang Uygur Autonomous Region, Yunnan Province, and Gansu Province, respectively [51]. However, in this study, *A. phagocytophilum* and *A. bovis* 16S rRNA gene sequences and *A. ovis* msp4 gene sequences were studied in Tan sheep in Ningxia, China, and *A. phagocytophilum* and *A. ovis* could be detected, but *A. bovis* was not detected. In addition, the msp4 gene of *A. ovis* was detected to be similar to the “sheepXJ2” and “sheepXJ9” strains isolated from sheep in southern Xinjiang [52], indicating that they belong to the same clade as the sheep isolated from southern Xinjiang. *A. phagocytophilum* showed high homology with Jilin, Gansu, Turkey, and Japan isolates. There are many reasons for this situation, which may be a pathogen import phenomenon caused by the cross-border migration of vector ticks or live animals or animals, and the cross-regional transport of livestock products. Most importantly, humans and all kinds of animals are generally susceptible to anaplasmosis. Since the disease was first identified in the United States in 1994, *A. phagocytophilum* has also been detected in many countries. Ticks play an irreplaceable role in the transmission of this disease. Eliminating ticks and reducing contact with ticks play a key role in the prevention and treatment of this disease. Ningxia is located in northwest China, with a diverse ecological environment and a wide range of bacteria carrying animals and ticks. Herdsmen, veterinary workers, and mountaineers must prevent tick bites to reduce the risk of infection when they are out and about.

From the results of the present study, *Anaplasma* spp. appear to be more harmful than *Theileria* and *Babesia*, not only because it has the highest infection rate, but also because it is highly infectious to humans. It can cause fever, emaciation, leukopenia and thrombocytopenia, and multiorgan failure in the host. Health policy makers, veterinarians, and farmers can consider this information for scientific management of Tan sheep in Ningxia. At the same time, in view of the fact that the number of pure Tan sheep breeding scale is relatively small, breeding requirements are high, and cross with other breeds of sheep will not occur, so the collected Tan sheep samples are very pure, so the detection rate of *Piroplasmids* and *Anaplasma* spp. is relatively accurate in Ningxia. In addition, the limitation of this study is that the risk factors associated with this infection were not analyzed at the animal or farm level, which may be more helpful in developing optimal preventive measures by identifying the potential risk factors associated with tick-borne diseases, and therefore, further studies by a large number of researchers are needed.

## 5. Conclusions

This study is the first to reveal the incidence of *T. uilenbergi*, *B. ovis*, *B. motasi*, *A. ovis*, and *A. phagocytophilum* and the total prevalence of *Piroplasmids* and *Anaplasma* spp. in sheep in Ningxia, China. The infection rates of *Theileria*, *Babesia*,and *Anaplasma* were 20.7%, 12.7%, and 28.0%, respectively. The dominant parasite species were *T. uilenbergi* and *A. ovis*. The genotypes of the four detected parasites were similar to those found both domestically and internationally, with some genotypes unique to Ningxia and others similar to those observed elsewhere. Our results provide basic data for understanding the molecular epidemiology of *Piroplasmids* and *Anaplasma* spp. in Tan sheep in Ningxia and lay a theoretical foundation for the prevention and control of *Piroplasmids* and *Anaplasma* spp. in Tan sheep in Ningxia.

## Figures and Tables

**Table 1:** Primer sequences.

Name	Primers (5′−3′)	Annealing temperature (°C)	PCR products (bp)	References
*T. luwenshuni*18S-F	CATCTTCTTTTTGATGAGTTGA	57	1,123	Nijhof et al. [23]
*T. luwenshuni*18S-R	GCTTGATCCTTCTGCAGGTTCA			
*T. uilenbergi*18S-F	AAGTGAATAGCGAGATGG	55	1,619	
*T. uilenbergi*18S-R	TTAGGGAAAGTAAAGGTG			
*T. ovis*18S-F	TTTTGCTCCTTTACGAGTCTTT	52	904	
*T. ovis*18S-R	TCGTTCACGATTAATAATTGCA			
*B. motasi*18S-F	TTTGCGATGTTCCATTCA	52	1,489	This study
*B. motasi*18S-R	CACCTACGGAAACCTTGT			
*B. ovis*18S-F	TGATCCTGCCAGTAGTCAT	62	1,650	
*B. ovis*18S-R	CACCTACGGAAACCTTGT			
*A. phagocytophilum*16S-F	TGGGATAGCCACTAGAAAT	55	1,297	Barlough et al. [24]
*A. phagocytophilum*16S-R	GTACAAGACCCGAGAACG			
*A. bovis*16S-F	CTCGTAGCTTGCTATGAGAAC	55	551	
*A. bovis*16S-R	TCTCCCGGACTCCAGTCTG			
*A. ovis* msp4-F	GGGAGCTCCTATGAATTACAGAGAATTGTTTAC	54	870	De La Fuente et al. [25]
*A. ovis* msp4-R	CCGGATCCTTAGCTGAACAGGAATCTTGC			

**Table 2:** Test results of samples from different regions.

District	Number of samples (s)	Sample positive rate			
		*Theileria* spp.	*Babesia* spp.	*Anaplasma* spp.	Mixed infection
Yanchi County	30	30.0% (10/30)	16.7% (5/30)	46.7% (14/30)	6.0% (2/30)
Haiyuan County	30	13.3% (4/30)	0 (0/30)	26.7% (8/30)	3.3% (1/30)
Tongxin County	30	6.0% (2/30)	0 (0/30)	20% (6/30)	0 (0/30)
Lingwu City	30	23.3% (7/30)	16.7% (5/30)	3.3% (1/30)	3.3% (1/30)
Hongsibu	30	26.7% (8/30)	30% (9/30)	43.3% (13/30)	10% (3/30)
Total	150	20.7% (31/150)	12.7% (19/150)	28.0% (42/150)	4.7% (7/150)

**Table 3:** Detection results of different species of *Piroplasmids* and *Anaplasma* spp. in different regions.

District	Number of samples (s)	Sample positive rate							
		*T. uilenbergi*	*T. luwenshuni*	*T. ovis*	*B. ovis*	*B. motasi*	*A. ovis*	*A. phagocytophilum*	*A. bovis*
Yanchi County	30	33.3% (10/30)	0 (0/30)	0 (0/30)	10.0% (3/30)	6.0% (2/30)	33.3% (10/30)	13.3% (4/30)	0 (0/30)
Haiyuan County	30	13.3% (4/30)	0 (0/30)	0 (0/30)	0 (0/30)	0 (0/30)	20% (6/30)	6.7% (2/30)	0 (0/30)
Tongxin County	30	6.0% (2/30)	0 (0/30)	0 (0/30)	0 (0/30)	0 (0/30)	16.7% (5/30)	3.3% (1/30)	0 (0/30)
Lingwu City	30	23.3% (7/30)	0 (0/30)	0 (0/30)	16.7% (5/30)	0 (0/30)	0 (0/30)	3.3% (1/30)	0 (0/30)
Hongsibu	30	26.7% (8/30)	0 (0/30)	0 (0/30)	6.7% (2/30)	23.3% (7/30)	40.0% (12/30)	3.3% (1/30)	0 (0/30)
Total	150	20.7% (31/150)	0 (0/150)	0 (0/150)	6.7% (10/150)	6.0% (9/150)	22.0% (33/150)	6.0% (9/150)	0 (0/150)

**Table 4:** Test results of samples of different ages.

Age (months)	Number of samples (s)	Sample positive rate		
		*Theileria* spp.	*Babesia* spp.	*Anaplasma* spp.
20–30	55	25.4% (14/55)^A^	23.6% (13/55)^A^	36.3% (20/55)^A^
30–40	49	12.2% (6/49)^B^	6.1% (3/49)^B^	18.3% (9/49)^B^
40–50	46	23.9% (11/46)^A^	6.5% (3/46)^B^	28.2% (13/46)^A^
Total	150	20.7% (31/150)	12.7% (19/150)	28.0% (42/150)

*Note*. The difference between different letters in the same column is significant (*P*  < 0.05); the difference between the same letter is not significant (*P*  > 0.05).

## Data Availability

The nucleotide sequences obtained in this study were deposited in the GenBank database. The accession numbers were *T. uilenbergi* (OR739069–OR739073); *Babesia* spp. (OR735152–OR735157); *A. phagocytophilum* (OR735468–OR735472); and OR766697–OR766704 of *A. ovis* msp4 gene in sheep.
